# miR-200c Modulates the Pathogenesis of Radiation-Induced Oral Mucositis

**DOI:** 10.1155/2019/2352079

**Published:** 2019-06-27

**Authors:** Jingjing Tao, Mengjing Fan, Difan Zhou, Yiyang Hong, Jing Zhang, Hai Liu, Sherven Sharma, Guanyu Wang, Qinghua Dong

**Affiliations:** ^1^Biomedical Research Center, Sir Run Run Shaw Hospital, School of Medicine, Zhejiang University, Hangzhou, Zhejiang, China; ^2^Department of General Surgery, Sir Run Run Shaw Hospital, School of Medicine, Zhejiang University, Hangzhou, Zhejiang, China; ^3^Department of Radiotherapy, Sir Run Run Shaw Hospital, School of Medicine, Zhejiang University, Hangzhou, Zhejiang, China; ^4^David Geffen School of Medicine at UCLA and the Veterans Affairs, Los Angeles, CA, USA; ^5^Key Laboratory of Cancer Prevention and Intervention, China National Ministry of Education, China

## Abstract

Radiation-induced oral mucositis (RIOM) is one of the most common side effects of radiotherapy in cancer patients, especially in almost all head and neck cancer patients. It presents as severe pain and ulceration. The development of RIOM is composed of five stages: initiation, primary damage response, signal amplification, ulceration, and healing. However, the key regulators involved in the RIOM pathogenesis remain largely unknown. In this study, we reveal a novel role of miR-200c, a member of the miR-200 family, in modulating RIOM pathogenesis. Using a mouse model mimicking RIOM, we found that the miR-200 family numbers (miR-141, miR-200a, miR-200b, and miR-200c) except miR-429 were significantly induced during the RIOM formation. Besides, in RIOM mice, miR-200c expression level was also increased dramatically in the normal human keratinocytes (NHKs) after irradiation. Knockdown of miR-200c expression with miR-200c-3p-shRNA significantly reduced senescence phenotype and enhanced cell proliferation in NHKs after irradiation. The generation of reactive oxygen species (ROS) and p47 enzyme involved in ROS production was increased after irradiation but both were markedly reduced in NHKs by miR-200c inhibition. Knockdown of miR-200c expression in NHKs increased DNA double-strand break repair after irradiation compared with control NHKs. Furthermore, miR-200c inhibition repressed the production of proinflammatory cytokines (TGF-*β*, TNF-*α*, and IL-1*α*) via inhibiting NF-*κ*B and Smad2 activation in NHKs exposed to IR. Additionally, miR-200c inhibition promoted NHK migration and increased the expression of molecules that regulate epithelial to mesenchymal transition, including Snail, Vimentin, Zeb1, and Bmi-1. These results not only identify the key role of miR-200c in the pathogenesis of RIOM but also provide a novel therapeutic target to treat RIOM.

## 1. Introduction

Radiation-induced oral mucositis (RIOM) is one of the main adverse events of radiotherapy (RT). Approximately 80–100% of patients receiving RT for head and neck cancer develop RIOM. RIOM is characterized by ulceration and results in severe pain, interference in treatment administration, and can even worsen the quality of life, also affects patient survival [1]. Though the key regulators involved in RIOM pathogenesis remain largely unknown, a five-stage model was proposed to explain the development of RIOM: initiation, primary damage response, signal amplification, ulceration, and healing [2]. IR injury results in lethal DNA damage (mainly DNA double-strand breaks, DSB) with generation of reactive oxygen species (ROS). This is followed by NF-*κ*B activation, releasing large amounts of proinflammatory cytokines [3]. These cytokines not only damage the tissue but also provide a positive-feedback loop to amplify the primary damage initiated by radiation. For example, TNF-*α* acts through the TNF-*α* receptor family and can activate NF-*κ*B signaling again. Then, the mucosal ulcerations form. During the last healing stage, the basal epithelial cells can migrate, proliferate, and differentiate to heal the ulcer. Traditional treatments, such as pain control, nutritional support, antibiotic administration, or immune modulation, targeting a single pathogenic process, may reduce symptoms of RIOM, but these treatments are not sufficient to cure the disease and also have various side effects [4]. Therefore, it is essential to identify the key regulators that control the pathogenic processes of RIOM and to develop alternative therapeutic strategies to treat RIOM.

MicroRNAs are small noncoding RNAs that regulate posttranscriptionally by mRNA cleavage or translational repression [5]. Because a single miRNA targets hundreds of mRNAs, miRNA plays an important regulatory role in the initiation of various diseases. The miR-200 family contains five homologous miRNAs including miR-200a, miR-200b, miR-200c, miR-141, and miR-429. Extensive research have demonstrated that miR-200 family inhibits the epithelial-to-mesenchymal transition (EMT) and tumor metastasis, represses cancer stem cell self-renewal, reverses chemoresistance, and enhances radiosensitivity in some types of cancer [6]. It has previously been observed that miR-200 family is involved in the response to oxidative stress across multiple species [7]. miR-200c overexpression regulates the oxidative stress response genes and increases cellular radiosensitivity in lung cancer cells [6]. Nonetheless, the function of miR-200c in the regulation of RIOM is still unclear.

Bmi-1 and Zeb1 are two key genes targeted by miR-200c and miR-141 [8]. Bmi-1 is known to mitigate IR-induced genotoxicity and protects normal human keratinocytes (NHKs) after IR [9]. Zeb1 is a crucial regulator in EMT which promotes wound healing [10]. Recent study showed that increased miR-200c expression in the skin is related to age-associated delay in wound healing and compromises skin repair in chronic wounds [11]. Thus, we postulated that miR-200c could modulate the RIOM pathogenesis. In this study, we investigated the role of miR-200c in modulating the pathogenesis of RIOM and found that miR-200a, miR-200b, miR-200c, and miR-141 were induced during RIOM in the mouse model. We also identified that the most significantly expressed miR-200 was miR-200c in normal NHKs after IR. We further demonstrated that miR-200c inhibition decreased the IR-induced senescence, ROS generation, and production of proinflammatory cytokines, molecularly through inhibiting p16, p47 expression and activating Smad2 and NF-*κ*B signaling. Meanwhile, miR-200c inhibition enhanced cell proliferation and DNA damage repair and promoted cell wound healing ability and EMT-related gene expressions. Collectively, our studies not only identify the novel functional role of miR-200c in modulating pathogenic processes of RIOM but also provide a potential therapeutic target for RIOM treatment.

## 2. Materials and Methods

### 2.1. Radiation-Induced Oral Mucositis Mouse Model

All animal care and experimental procedures strictly followed the recommendations in the Guide for the Care and Use of Laboratory Animals of Zhejiang University and were approved by the Committee on the Ethics of Animal Experiments of Zhejiang University. We developed the RIOM mouse model by exposing the head and neck area of C57/BL6 mice (6-8 weeks old) to radiation from X-RAD 160 (Precision X-ray, New Haven, CT) with a lead shield covering the rest of the body [12]. We treated the mice with different single doses (15, 18, 20, 25, and 30 Gy) in preliminary experiments and selected 25 Gy as the optimal dosage to rapidly induce radiogenic mucositis. Mice were anesthetized during irradiation. At 7 days posttreatment, the excised tongues were stained with 1% toluidine blue (TB) in 10% acetic acid till there were no further staining and washed with 1% acetic acid [13]. Increased TB staining and a change in epithelial thickness were used as markers of oral mucositis. At 3, 4, 5, 6, and 7 days posttreatment, the tongues (*n* = 5 in each group) were frozen at -80°C and used for qPCR analysis for miR-200 family and inflammation cytokines.

### 2.2. Immunohistochemistry (IHC) and Hematoxylin-Eosin (H&E) Staining

The tongues were fixed with 4% paraformaldehyde and embedded in paraffin, which was used in 5 *μ*m sections to conduct histologic analysis. For H&E staining, tissue samples were sectioned from the middle of the wounds, then stained with hematoxylin and eosin. In order to visualize Bmi-1 and Zeb1 in the tongues, standard immunoperoxidase procedures were used and the sections from the tongues (*n* = 5 in each group) were incubated with primary antibodies overnight at 4°C, then were further incubated with horseradish peroxidase-linked secondary antibodies for 2 hours at room temperature.

### 2.3. Cell Culture

This study was performed strictly according to the recommendations from the Guide for Clinical Research which were provided by Sir Run Run Shaw Hospital, Zhejiang University. This protocol was approved and monitored by the Ethics Committee of Sir Run Run Shaw Hospital, Zhejiang University (No. 20170222-26). Informed consent was obtained from all patients. Primary NHKs were isolated from human foreskins according to protocols described elsewhere [14]. NHKs were cultured in serum-free EpiLife™ medium with calcium supplied with defined growth supplement (Invitrogen, Carlsbad, CA). To generate NHK/Control and NHK/miR-200c- cells, NHKs were infected with lentivirus carrying control vector (hU6-MCS-CMV-EGFP) or miR-200c-3p-shRNA (Genechem, Shanghai) and further selected by puromycin. To generate the irradiation experiments, NHKs were exposed to varying irradiation doses with X-RAD 160 irradiator.

### 2.4. Clone Formation Assay

NHKs were cultured at 6-well plates with 1000-6000 cells per well and treated correspondently with 0, 4, 6, 8, and 10 Gy irradiation. Cells were further cultured for 10 days after irradiation. 0.5% crystal violet was used for staining, then the colonies were counted. The surviving fraction was calculated by the proportion of seeded cells after being irradiated to form colonies relative to untreated cells. To further calculate the cellular radiosensitivity (mean lethal dose, *D*_0_) and the capacity for sublethal damage repair (quasithreshold dose, Dq), the equation SF = 1 − (1 − *e* ^−*D*/*D*_0_^)^*N*^ was applied. Those values were then used to calculate the sensitization enhancement ratio (SER) [15].

### 2.5. Senescence-Associated *β* Galactosidase (SA-*β*-Gal) Activity Assay

After being washed in PBS twice, NHKs were further fixed in 2% formaldehyde, 0.2% glutaraldehyde solution. SA-*β*-Gal staining solution (Beyond Biotech, China) was used for staining, then incubated at 37°C for 16 h.

### 2.6. Western Blot Analysis and Immunofluorescence Staining

Protein extraction and the assay were carried out as described in our previous study [9]. The antibodies used in this study are listed in Table S1.

For immunofluorescence staining, we fixed cells in 4% paraformaldehyde at room temperature for 15 min, then permeabilized in 0.25% TritonX-100 for 15 min, blocked with 5% goat serum for 1 hour. We used mouse monoclonal anti-phospho-*γ*-H2AX and Alex Fluor®549 goat anti-mouse IgG (Thermo Fisher A-11005) as primary and secondary antibodies. Nuclei were stained with DAPI in SlowFade® Gold Antifade Mountant (Thermo Fisher, S36942). Images were captured with a Zeiss HBO-100 fluorescence microscope (Carl Zeiss, Germany).

### 2.7. Reverse Transcription qPCR

Total RNA were extracted by using TRIzol™ Reagent (Invitrogen™). Reverse transcription was performed with a reverse transcription system (Promega, Madison, WI, USA) according to the manufacturer's instructions. qPCR was carried out three times for each sample using FastStart Universal SYBR Green Master (Roche Diagnostics, Rotkreuz, Switzerland) to determine the mRNA level of cytokines. To detect mir-200 family expression, All-in-One miRNA RT-PCR Detection Kit (GeneCopoeia, Rockville, MD) was used according to the manufacturer's instructions. Reverse transcription reaction was performed with All-in-One cDNA Synthesis SuperMix (Bimake, B24403); qPCR was performed using SYBR Green qPCR Master Mix (Bimake, B21202). Primer sequences are listed in Table S2.

### 2.8. Determination of Intracellular ROS Level

NHKs were stained with 5 *μ*M dihydroethidium (DHE) (Beyotime Biotech, China) at room temperature for 30 min. We use FACSCalibur using CellQuest software (Becton Dickinson, San Jose, CA) to quantify fluorescence intensity of DHE.

### 2.9. Neutral Comet Assay

A neutral comet assay was performed to detect the level of DNA DSBs, using the CometAssay Kit (Trevigen, Gaithersburg, MD) [16]. After being exposed to 5 Gy IR, cells were harvested at 10 h after irradiation and were further electrophoresed in the CometSlides. GelRed was used for staining. Images were obtained by the Zeiss HBO-100 fluorescence microscope. Degree of DNA damage was compared in the groups by measuring the DNA containing ratio of head/tail using comet assay software project (CASP). The threshold of CASP parameters was adjusted: head center threshold (HCT) = 0.8, tail threshold (TT) = 0.05, head threshold (HT) = 0.05, and comet threshold (CT) = 0.05. The tail DNA was measured by the sum of intensities of pixels in the tail according to the online protocol.

### 2.10. Wound Healing

NHKs were seeded in 6-well plates (5 × 10^5^ cells/well) until they reached 90% confluence, a linear wound was created by scratching using the pipette tip [17]. After 5 Gy IR, photos were taken at 0, 24, and 30 hours.

### 2.11. Statistical Analysis

To detect the differences among various treatments, one-way analysis of variance (ANOVA) was used; the paired Student's *t*-test was used for evaluation of the differences between two groups. Differences with *P* < 0.05 were considered significant.

## 3. Results

### 3.1. miR-200 Family Numbers Are Induced during RIOM

**Figure 1:**
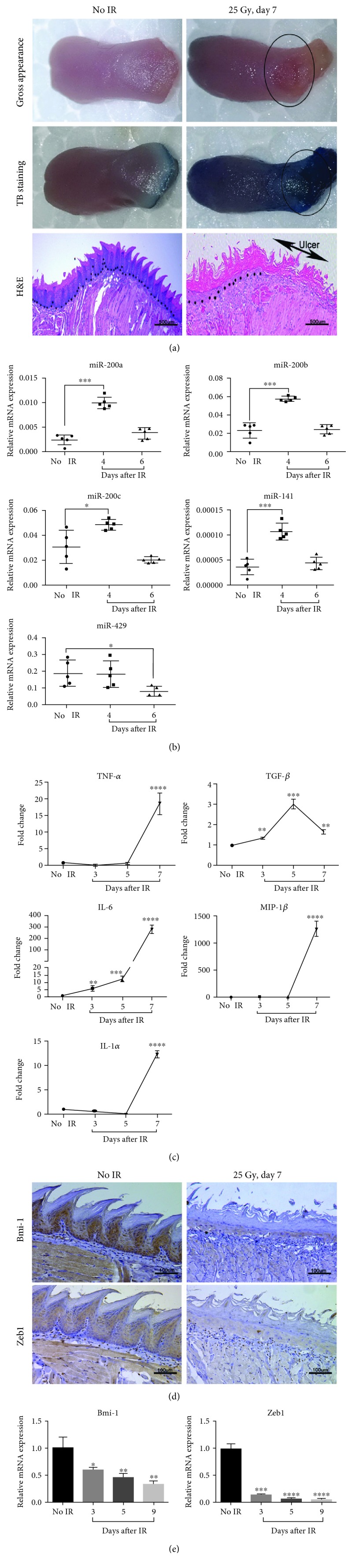
miR-200 family numbers are induced during RIOM. (a) Representative images of RIOM gross appearance, toluidine blue (TB) staining, and HE staining of tongue tissues from nonirradiated and irradiated C57BL/6 mice at day 7 after 25 Gy IR. Arrows show the areas of ulcer. The dot lines refer to epithelial-stromal boundary. (b) The tongues of C57BL/6 mice were harvested at days 0, 4, and 6 postirradiation; the expression level of miR-200 family was measured by qPCR (*n* = 5 per group). ^∗^ *P* < 0.05 and ^∗∗∗^ *P* < 0.001 vs. nonirradiated control mice. (c) The tongues of C57BL/6 mice were harvested at days 0, 3, 5, and 7 postirradiation, then the mRNA levels of TNF-*α*, TGF-*β*, IL-6, IL-1*α*, and MIP-1*β* were measured by qPCR (mean ± S.D.). The proinflammatory cytokines were induced during RIOM formation. ^∗∗^ *P* < 0.01, ^∗∗∗^ *P* < 0.001, and ^∗∗∗∗^ *P* < 0.0001 vs. nonirradiated control mice. (d) IHC staining of Bmi-1 and Zeb1 of tongue tissues from nonirradiated and irradiated mice at day 7 after 25 Gy IR. (e) The mRNA level of Bmi-1 and Zeb1 in the tongues of C57BL/6 mice harvested at days 0, 3, 5, and 9 postirradiation was analyzed by qPCR. ^∗^ *P* < 0.05, ^∗∗^ *P* < 0.01, ^∗∗∗^ *P* < 0.001, and ^∗∗∗∗^ *P* < 0.0001 vs. nonirradiated control mice.

To determine if miR-200 family was involved in RIOM, we generated the RIOM mouse model by exposing the mouse to 25 Gy irradiation. Ulcers were seen at the posterior surface of the tongue, and the TB-stained ulcers were evident at day 7 after irradiation. Histological analyses showed complete depletion of the stratified squamous keratinized epithelium (Figure 1(a)). We checked miR-200 family expression in the tongues at days 4 and 6 (during RIOM ulcer formation) after irradiation. Most members of miR-200s (miR-141, miR-200a, miR-200b, and miR-200c) were significantly increased at day 4 after irradiation compared to the nonirradiated group, then decreased to the normal level at day 6 after irradiation. Only miR-429 expression level did not change at day 4 but decreased obviously at day 6 after irradiation (Figure 1(b)).

Cytokine-induced excessive inflammation is the key feature of RIOM [18]. We thus investigated the expression levels of 5 proinflammatory cytokines in the tissue of oral mucositis at different time points after irradiation. TNF-*α*, MIP-1*β*, and IL-1*α* were dramatically increased at day 7 after irradiation. IL-6 was increased obviously from days 3, 5, and 7 after irradiation. TGF-*β* was increased from day 3 to day 5 but decreased at day 7 after irradiation (Figure 1(c)). Consistent with the increased miR-200s, mRNA and protein expression levels of their target genes Bmi-1 and Zeb1 were substantially reduced in RIOM tongues (Figures 1(d) and 1(e)). These data indicate that RIOM is associated with the miR-200s expression.

### 3.2. miR-200c Modulates Proliferation and Senescence in NHKs Exposed to IR

**Figure 2:**
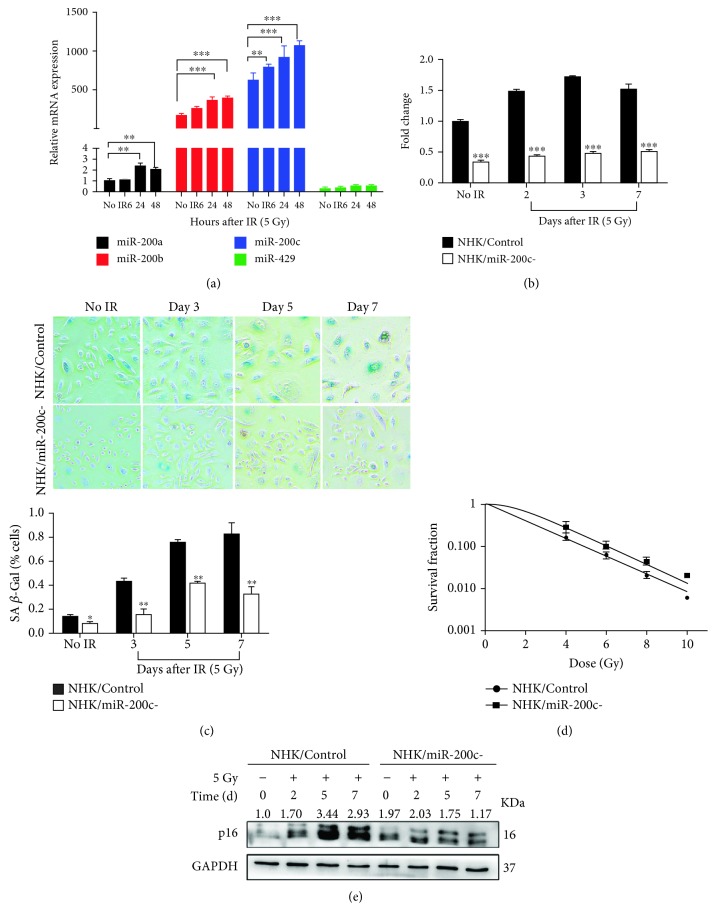
miR-200c modulates proliferation and senescence in NHKs exposed to IR. (a) The expression level of miR-200 family was measured by qPCR (mean ± S.D.) at indicated time after being exposed to 5 Gy. 5S rRNA was used as an internal control. ^∗∗^ *P* < 0.01 and ^∗∗∗^ *P* < 0.001 vs. nonirradiated cells. (b) NHKs were infected with sh-miR-200c (NHK/miR-200c-) or a scrambled negative control (NHK/Control); miR-200c expression was measured by qPCR (mean ± S.D.) at days 2, 3, and 7 after being exposed to 5 Gy. 5S rRNA was used as an internal control. ^∗∗∗^ *P* < 0.001 vs. NHK/Control. (c) SA-*β*-Gal assay was performed in NHK/Control and NHK/miR-200c- cells at days 0, 3, 5, and 7 postirradiation; the numbers of positive cells were counted (mean ± S.D.). IR-induced senescent cells were much less in NHK/miR-200c- compared with NHK/Control cells. ^∗^ *P* < 0.05 and ^∗∗^ *P* < 0.01 vs. NHK/Control. Scale bar 200 *μ*m. (d) NHK/Control and NHK/miR-200c- cells were cultured in 6-well plates for 24 hours before being irradiated with the indicated doses. Cells were further cultured for 10 days, then the colonies were counted; surviving fractions were determined by the number of colonies divided by the number of seeded cells × plating efficiency. (e) Western blotting was performed for p16^INK4A^ and GAPDH (internal control) in NHK/Control and NHK/miR-200c- cells at indicated time after being exposed to 5 Gy.

To investigate if the miR-200 family was also upregulated by IR *in vitro*, NHKs were exposed to 5 Gy IR. miR-141 was undetectable in NHKs, and miR-429 expression had no change compared with nonirradiated cells. However, miR-200a, miR-200b, and miR-200c were increased significantly even at 48 h after irradiation (Figure 2(a)). Among them, miR-200c was the most significantly induced miR-200 in NHKs and increased as early as 6 h after irradiation. Therefore, we selected miR-200c for our following investigations. To determine if miR-200c contributes to RIOM in NHKs, we generated miR-200c knockdown NHKs (NHK/miR-200c-) by infecting lentiviruses encoding miR-200c-specific shRNA sequence. miR-200c expression increased dramatically at days 2, 3, and 7 after IR in the NHK/Control cells, but it was inhibited in the NHK/miR-200c- cells (Figure 2(b)). Previous study has demonstrated that stress-induced premature senescence is the main cellular response of NHKs to irradiation [9]. We found that the knockdown of miR-200c notably suppressed SA-*β*-gal activity in irradiated NHKs. Furthermore, the NHK/miR-200c- cells maintained being active for cell proliferation when NHK/Control cells became nonreplicative at day 7 after IR (Figure 2(c)). Radiation response of NHKs was further evaluated with the colony-forming assay, a gold standard for determining radiosensitivity [19]. As expected, we observed that the ability of clone formation and the plating efficiency of NHK/miR-200c- cells were much higher than those of NHK/Control cells after IR (Figure 2(d)). miR-200c inhibition decreased the radiosensitivity of NHK, with a SER of 0.23 (Table S3). In addition, miR-200c inhibition notably suppressed IR-induced expression of cell cycle molecule p16^INK4A^, which mediate telomere-independent senescence (Figure 2(e)). Thus, miR-200c inhibition promoted cell proliferation while it prevented senescence of NHKs after IR. These data support our hypothesis that miR-200c may modulate the pathogenesis of RIOM.

### 3.3. miR-200c Regulates ROS Generation and DNA Repair in NHKs after Irradiation

**Figure 3:**
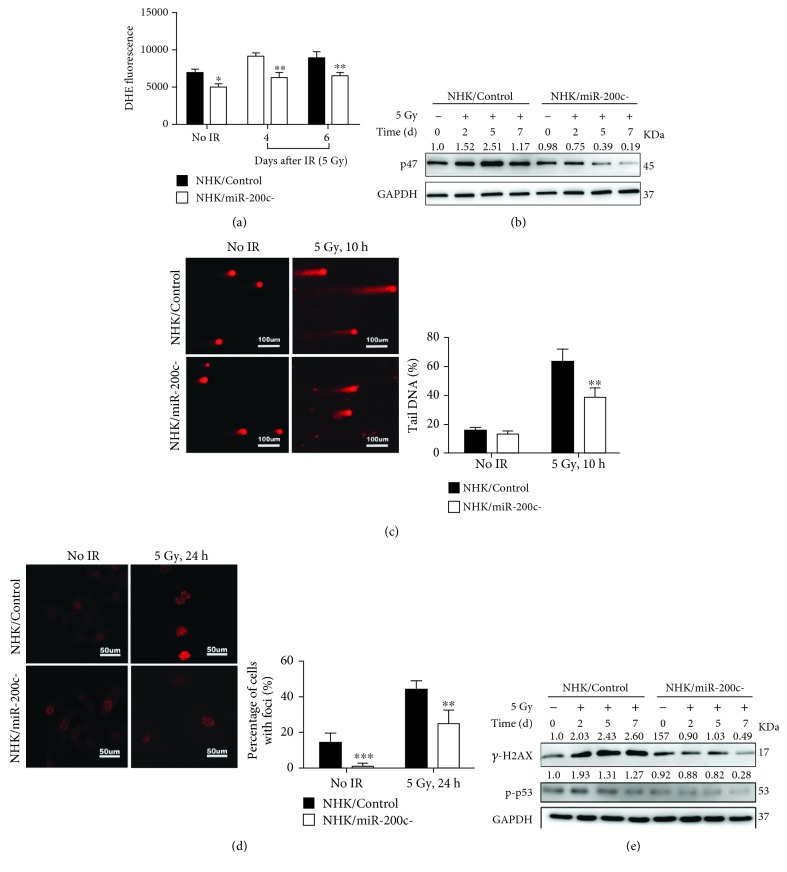
miR-200c regulates ROS generation and DNA repair in NHKs after irradiation. (a) NHK/Control and NHK/miR-200c- cells were exposed to 5 Gy IR and stained with DHE. Fluorescence intensity was quantitatively determined by flow cytometry. ^∗^ *P* < 0.05 and ^∗∗^ *P* < 0.01 vs. NHK/Control cells. (b) Western blotting was performed for p47 and GAPDH (internal control) in NHK/Control and NHK/miR-200c- cells at indicated time after being exposed to 5 Gy. (c) NHK/Control and NHK/miR-200c- cells were exposed to 5 Gy radiation and neutral comet assay was performed. Photos were taken at 0 and 10 h postirradiation, and DNA DSB was quantitated by DNA containing ratio of head/tail (mean ± S.D.). ^∗∗^ *P* < 0.01 vs. NHK/Control cells. (d) NHK/Control and NHK/miR-200c- cells were irradiated at 5 Gy; cells were further stained with the *γ*-H2AX antibody 24 h after radiation. Representative images were taken; the percentage of *γ*-H2AX foci positive cells (>5 intranuclear foci) was statistically analyzed (mean ± S.D.). ^∗∗^ *P* < 0.01 and ^∗∗∗^ *P* < 0.001 vs. NHK/Control cells. (e) Western blotting was performed for *γ*-H2AX, p-p53, and GAPDH (internal control) in NHK/Control and NHK/miR-200c- cells at indicated time after being exposed to 5 Gy.

In the initiation stage of RIOM, radiotherapy induces a direct and lethal DNA damage and ROS generation in epithelial cells. We first investigated the effect of miR-200c on ROS generation. DHE staining showed that ROS was obviously generated in the NHK/Control cells at 4 and 6 days after irradiation, but it was markedly suppressed in the NHK/miR-200c- cells (Figure 3(a)). Accordingly, p47-phox, an important enzyme involved in ROS generation, was increased in the NHK/Control cells at 2, 5, and 7 days post IR, but the induction was inhibited in the NHK/miR-200c- cells (Figure 3(b)).

Then, we further determined whether miR-200c inhibition affects DNA damage response in NHKs by the comet assay and *γ*-H2AX staining, which are the standard methods to detect DSB formation. NHK/miR-200c- cells exhibited shorter comet tails and less DNA containing ratio of head/tail than NHK/Control cells at 10 hours after irradiation, suggesting less DSB formation (Figure 3(c)). Furthermore, irradiation notably increased *γ*-H2AX foci formation in the NHK/Control cells, whereas the formation level was markedly reduced in NHK/miR-200c- cells after irradiation (Figure 3(d)). Consistent to the *γ*-H2AX foci formation, the protein levels of *γ*-H2AX and p-p53 in NHK/miR-200c- cells were markedly reduced after IR compared with NHK/Control cells (Figure 3(e)). These data indicate that miR-200c inhibition mitigates the genotoxic effects of IR by suppressing ROS generation and enhancing repair of damaged DNA.

### 3.4. miR-200c Regulates Proinflammatory Cytokine Induction and TGF-*β*/NF-*κ*B Activation in NHKs after Irradiation

**Figure 4:**
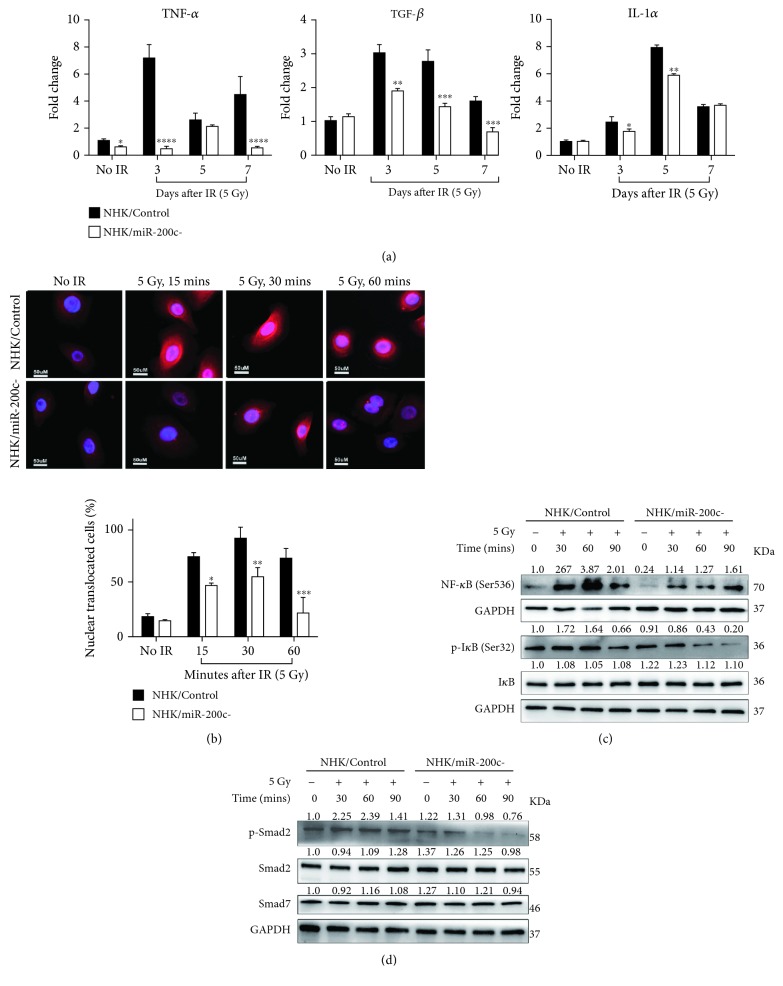
miR-200c regulates proinflammatory cytokine induction and TGF-*β*/NF-*κ*B activation in NHKs after irradiation. (a) NHK/Control and NHK/miR-200c- cells were exposed to 5 Gy IR; the expression of TNF-*α*, TGF-*β*, and IL-1*α* was measured by qPCR at 0, 3, 5, and 7 days postirradiation. ^∗^ *P* < 0.05, ^∗∗^ *P* < 0.01, and ^∗∗∗^ *P* < 0.001 vs. NHK/Control cells. (b) NHK/Control and NHK/miR-200c- cells were exposed to 5 Gy radiation; the immunofluorescent images of NF-*κ*B (phosphor S 536) were taken at 0, 15, 30, and 60 min postirradiation. The NF-*κ*B nuclear translocated cell numbers were counted. (c) Western blotting was performed for NF-*κ*B (phosphor S536), I*κ*B (phosphor S32), I*κ*B, and GAPDH (internal control) in NHK/Control and NHK/miR-200c- cells at indicated time after being exposed to 5 Gy. (d) Western blotting was performed for p-Smad2, Smad2, Smad7, and GAPDH (internal control) in NHK/Control and NHK/miR-200c- cells at indicated time after being exposed to 5 Gy.

During the primary damage response stage of RIOM, NF-*κ*B is one of the most significantly activated pathways, which stimulates proinflammatory cytokine release from cells. We next determined the involvement of miR-200c in the regulation of cytokine release and NF-*κ*B activation induced by IR. We first evaluated the production of proinflammatory cytokines after irradiation by qPCR. TNF-*α*, TGF-*β*, and IL-1*α* were significantly induced in 5 Gy irradiated NHK/Control cells, but they were much less in the NHK/miR-200c- cells (Figure 4(a)).

We further examined IR-induced activation of NF-*κ*B by immunofluorescence staining. NF-*κ*B translocation into the nucleus was significantly increased as early as 15 minutes post IR and prolonged till 60 min in the NHK/Control cells. In contrast, the translocation of NF-*κ*B was reduced in the NHK/miR-200c- cells after IR (Figure 4(b)). Accordingly, IR rapidly increased levels of p-NF-*κ*B and p-I*κ*B in NHK/Control cells. miR-200c inhibition prevented the IR-induced NF-*κ*B and I*κ*B activation. The total I*κ*B levels had no significant change in both cells (Figure 4(c)). It has been reported that the NF-*κ*B-dependent gene expression in irradiated NHKs is dependent on the TGF-*β* pathway [20]. We then tested if miR-200c affects Smad-dependent TGF-*β* pathway. Phosphorylated Smad2 level was increased in irradiated NHK/Control cells, but it was diminished in NHK/miR-200c- cells (Figure 4(d)). We also checked the expression level of Smad7, a negative feedback inhibitor of TGF-*β*/Smad signaling, after IR. However, miR-200c inhibition had no effect on Smad7 level in both nonirradiated and irradiated NHKs (Figure 4(d)). These results indicate that miR-200c inhibition represses IR-induced production of proinflammatory cytokines through inhibiting NF-*κ*B and TGF-*β* signaling pathways.

### 3.5. miR-200c Regulates NHK Migration after Irradiation

**Figure 5:**
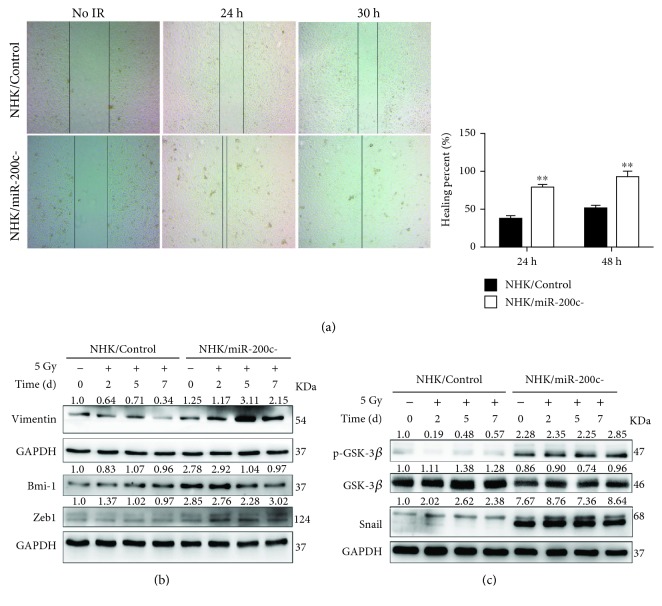
miR-200c regulates NHK migration after irradiation. (a) Wound healing assay was performed in NHK/Control and NHK/miR-200c- cells at indicated time after being exposed to 5 Gy. Left panel: images were taken at 0, 24, and 30 h after irradiation. Right panel: quantification of the healing percent (mean ± S.D.) of cells. ^∗∗^ *P* < 0.01 vs. NHK/Control cells. (b) Western blotting was performed for Vimentin, Zeb1, and Bmi-1 in NHK/Control and NHK/miR-200c- cells at indicated time after being exposed to 5 Gy. (c) Western blotting was performed for p-GSK-3*β*, GSK-3*β*, Snail, and GAPDH (internal control) in NHK/Control and NHK/miR-200c- cells at indicated time after being exposed to 5 Gy. miR-200c inhibition activated GSK-3*β*/Snail pathway.

Migrating keratinocytes promote the epithelium reepithelialization in the healing stage of RIOM. To test the effect of miR-200c in tissue healing, we examined the migration ability of NHKs after IR by the wound healing assay. miR-200c inhibition significantly promoted cell migration compared to the NHK/Control cells, and the healing percentage almost reached 100% at 30 hours after IR (Figure 5(a)). EMT is a key process in wound healing [21]. We then checked the expression levels of Zeb1 and Bmi-1, which are the target genes of miR-200c and can promote EMT. Bmi-1 level was much higher in NHK/miR-200c- cells compared with NHK/Control cells. Furthermore, miR-200c inhibition increased Zeb1 expression while it maintained Bmi-1 at a similar level after IR (Figure 5(b)). Meanwhile, Vimentin, a mesenchymal marker, was significantly increased in the irradiated NHK/miR-200c- cells. Given that GSK-3*β*/Snail signal pathway is importantly involved in modulating EMT process [21, 22], we further checked if miR-200c inhibition-induced EMT was also regulated by this pathway. miR-200c inhibition decreased the total amount of GSK-3*β* in irradiated NHKs, while it upregulated p-GSK-3*β* and Snail expression level in NHKs (Figure 5(c)). These results indicate that miR-200c inhibition promotes NHK migration through enhancing EMT.

## 4. Discussion

The pathobiology of RIOM is complex and multifaceted; thus, the key regulator of RIOM pathogenesis has not been fully understood. In this study, we show for the first time that miR-200c is the key regulator in modulating pathogenic processes of RIOM.

The pathogenesis of RIOM is similar in humans and mice. RIOM starts as an acute inflammation after RT exposure and lasts between 7 and 98 days in cancer patients [23, 24]. Using a mouse model mimicking RIOM, we found that miR-200s were significantly increased in tongue epithelium at day 4 after irradiation. Irradiated mice developed oral ulcer with epithelial ablation and overproduction of proinflammatory cytokines at day 7 post IR, consistent with the other reports [25]. Unlike in mice, miR-200c expression was the earliest and highest among miR-200s in NHKs. miR-200c inhibition protected NHKs from IR-induced senescence and maintained cell proliferation. These data demonstrated that miR-200c was involved in the pathogenesis of RIOM.

ROS is an important early trigger resulting in RIOM and in turn induced oxidative stress-associated DNA damage [26]. miR-200c inhibition reduced IR-induced cellular ROS generation and p47 protein expression. By analyzing *γ*-H2AX and comet assay, we found reduced DSB damage and more efficient repair of DNA in irradiated NHK/miR-200c- cells. Thus, miR-200c regulates ROS generation and DSB repair at the initiation stage of RIOM.

NF-*κ*B is one of the major signaling pathways activated by IR, which promotes proinflammatory cytokine (such as TGF-*β*) release from cells [27]. Signal amplification during RIOM is an important step in IR-induced injury; overproduced proinflammatory cytokines restimulate and amplify mucosal damage. In this study, miR-200c inhibition reduced NF-*κ*B nuclear translocation, I*κ*B activation, and proinflammatory cytokine production in irradiated NHKs, effectively alleviating inflammation. Besides NF-*κ*B, TGF-*β* signaling is also activated in oral mucositis [28]. TGF-*β* plays a key role in normal epidermal inflammatory responses; NF-*κ*B-dependent gene expression in keratinocytes after irradiation requires intact TGF-*β* signaling [20]. The key mediators in the TGF-*β* signaling pathway are the Smads [29]. Phosphorylated Smad2/3 is involved in the activation of TGF-*β* signaling. Smad7 is a negative feedback inhibitor which competes with Smad2/3 for binding to activated TGFR1 [30]. We found that IR-induced p-Smad2 was inhibited in NHK/mir-200c- cells, while Smad7 level was not changed. These results demonstrated that miR-200c inhibition reduced TGF-*β* activation through pSmad2 inhibition but not Smad7 promotion. Thus, miR-200c reduces inflammation by antagonizing NF-*κ*B and TGF-*β* activation.

Wound healing is associated with cellular proliferation, migration, and tissue remodeling [31]. In the healing stage of RIOM, keratinocytes migrate and proliferate to reepithelialize the epithelium [32]. EMT is a process required in wound healing after injury. Transcription factors Zeb1/2, Bmi-1, and Snail are master EMT regulators and also targets of miR-200c [33]. Zeb1 can efficiently inhibit the cell-cell adhesion molecule E-cadherin and promote EMT [34]. Bmi-1 can regulate Snail and promote EMT [35]. Studies show that inhibition of GSK-3*β* activity can subsequently suppress p-Snail and induce Snail protein nuclear localization, then promotes EMT [36, 37]. In this work, we showed that miR-200c inhibition increased the expression of p-GSK-3*β* and Snail in irradiated NHKs. Zeb1 and Bmi-1 expression levels were decreased in the tongue during RIOM. miR-200c inhibition increased Zeb1 and Bmi-1 levels in NHKs. Thus, miR-200c inhibition promotes NHK migration after IR through regulating EMT-related protein expressions and GSK-3*β*/Snail pathways.

Bmi-1 plays a role in some biological functions including senescence, self-renewal, DNA damage response (DDR), and cancer [38, 39]. Bmi-1 can maintain mitochondrial function and redox homeostasis [40]. Bmi-1 also protects NHKs from IR-induced DNA damage [9]. Bmi-1 is a crucial component in DDR, as it is required to recruit the DDR machinery to DSB sites after irradiation [40, 41]. Thus, the upregulation of Bmi-1 in irradiated NHK/miR-200c- cells may play a key role in reducing DNA damage and promoting DSB repair.

TGF-*β* promotes EMT in cancer cells [42], but it induces growth inhibition and apoptosis in keratinocytes. Knockdown of TGF-*β* signaling axis reduces skin scarring [43]. Bmi-1 reduces senescence of NHKs by inhibiting TGF-*β* signaling pathway [44]. Bmi-1 inhibits senescence and extends the lifespan of normal cells by suppressing p16^INK4A^ [45]. Thus, Bmi-1 may also play a key role in reducing senescence and promoting cell proliferation in irradiated NHK/miR-200c- cells.

**Figure 6:**
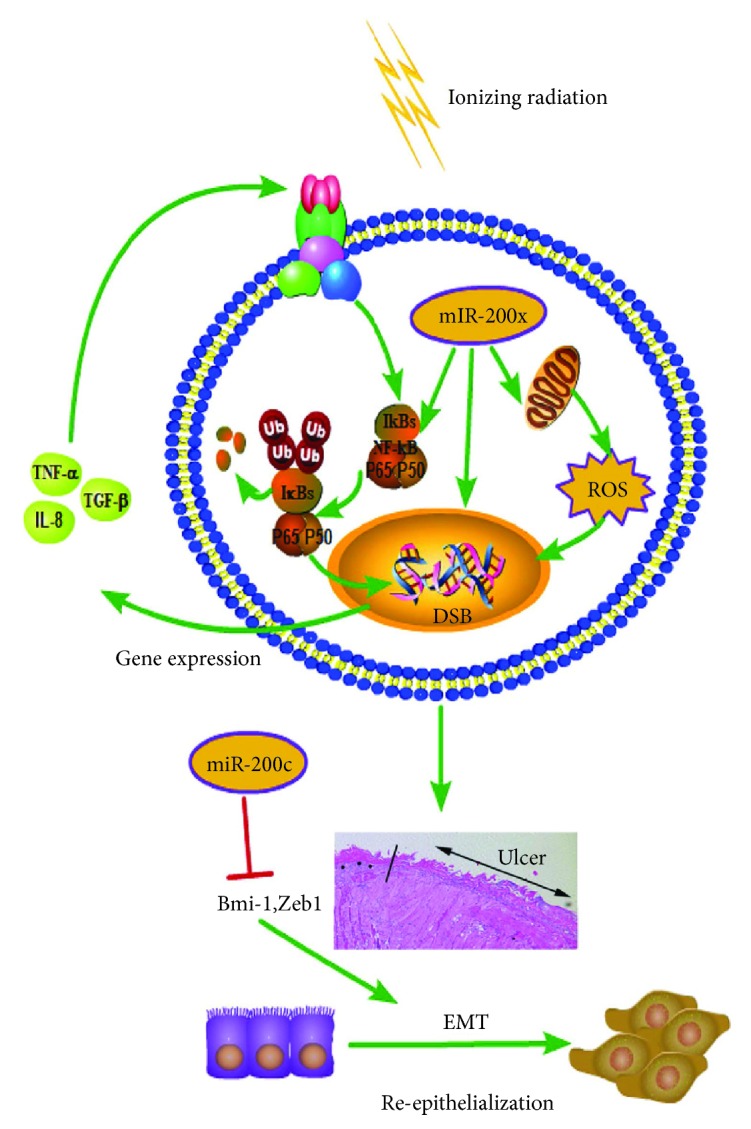
The potential role of miR-200c in regulating the pathogenesis of RIOM. miR-200c promoted radiation-induced NF-*κ*B signaling by increasing the phosphorylation level of I*κ*B*α* at the position ser32 and phosphorylation at position ser536 of p65 as well as nuclear translocation level, which led to overproduction of proinflammatory cytokines. miR-200c increased the DNA DSB level and ROS generation induced by radiation and caused more severe senescence phenotype. miR-200c decreased the expression of Bmi-1 and Zeb1, which led to downregulation of p-GSK-3*β* and Snail, thus inhibited NHK cell EMT. miR-200c mediated increased inflammation, DNA DSB, and ROS generation as well as decreased EMT capacity of NHK during the pathogenesis of RIOM.

In summary, we provide evidence that miR-200c modulates the pathogenesis of RIOM. Potential mechanisms for the context-specific effects of miR-200c are as follows: in the initiation stage of RIOM, miR-200c inhibition reduces radiation-induced ROS generation and DNA damage. In the primary damage response and signaling amplification stages, miR-200c inhibition attenuates TGF-*β*- and NF-*κ*B-mediated inflammation. In the healing stage, miR-200c inhibition promotes NHK migration through regulating the EMT process by upregulating its targets Zeb1and Bmi-1 and activating GSK-3*β*/Snail signal pathways (Figure 6). Bmi-1 may play the most important role in miR-200c-modulated pathogenesis of RIOM.

## Data Availability

The data used to support the findings of this study are available from the corresponding authors upon request.

## Abbreviations

DHE: Dihydroethidium
DSB: DNA double-strand break
EMT: Epithelial-mesenchymal transition
GAPDH: Glyceraldehyde-3-phosphate dehydrogenase
IR: Ionizing radiation
IL6: Interleukin 6
IL-1*α*: Interleukin-1*α*
NHK: Normal human keratinocyte
NF-*κ*B p65: Nuclear translocation factor 65 subunit
qPCR: Quantitative real-time polymerase chain reaction
RIOM: Radiation-induced oral mucositis
ROS: Reactive oxygen species
TGF-*β*: Transforming growth factor-*β*
TNF*α*: Tumor necrosis factor alpha.

## Supplementary Materials

Supplementary 1 Table S1: primary antibodies for western blot, IF, and IHC.

Supplementary 2 Table S2: primer sequences for quantitative RT-PCR.

Supplementary 3 Table S3: clonogenic survival parameters fitting the data to a multitarget model.
