# Supplementary material for: miR-200c Modulates the Pathogenesis of Radiation-Induced Oral Mucositis
Source: Oxid Med Cell Longev. 2019 Jun 27;2019:2352079. doi: 10.1155/2019/2352079 (PMC6620860; doi:10.1155/2019/2352079)
Supplement: Supplementary 1 — Table S1: primary antibodies for western blot, IF, and IHC. [file 2352079.f1.docx]

**Table S1:** Primary antibodies for western blot, IF and IHC.

| Protein | Name | Catalog number | Species | Mono or polyclonal | Dilution | Purpose |
| --- | --- | --- | --- | --- | --- | --- |
| Vimentin | Vimentin(D21H3) | 5741 (CST) | Rabbit | monoclonal | 1:1000 (WB) | WB |
| GAPDH | GAPDH | G5262 (Sigma) | Mouse | monoclonal | 1:1000 (WB) | WB |
| γ-H2AX | Anti-gamma H2AX (phospho S139) | Ab81299 (abcam) | Rabbit | monoclonal | 1:1000 (WB)  1:300 (IF) | IF  WB |
| p16 | Anti-p16 INK4a antibody | Ab54210  (abcam) | Mouse | monoclonal | 1:1000 (WB) | WB |
| p47 | P47 antibody | Sc-365215  (Santa Cruz Biotechnology) | Mouse | monoclonal | 1:1000 (WB) | WB |
| BMI-1 | Anti-Bmi-1 antibody | Ab38295  (abcam) | Rabbit | polyclonal | 1:300 (IHC)  1:1000 (WB) | WB  IHC |
| Zeb1 | Anti-zeb1-antibody | Ab87280  (abcam) | Rabbit | polyclonal | 1:150 (IHC)  1:1000 (WB) | WB  IHC |
| p-smad2 | Phospho-smad2 (Ser467) | 3108 (CST) | Rabbit | monoclonal | 1:1000 (WB) | WB |
| Smad2 | Smad2 (D43B4) | 5339 (CST) | Rabbit | monoclonal | 1:1000 (WB) | WB |
| Smad7 | Anti-Smad7 antibody | Ab216428 (abcam) | Rabbit | polyclonal | 1:1000 (WB) | WB |
| p-IκB | Anti-IκBα (phospho S32) antibody | Ab133462  (abcam) | Rabbit | monoclonal | 1:1000 (WB) | WB |
| IκB | Anti-IκBα antibody | Ab32518 (abcam) | Rabbit | monoclonal | 1:1000 (WB) | WB |
| NF-κB | Phospho-NF-κB p65 (Ser536) | 3036 (CST) | mouse | monoclonal | 1:1000 (WB)  1:100 (IF) | WB  IF |
| Snail | Snail (C15D3) | 3879 (CST) | Rabbit | monoclonal | 1:1000 (WB) | WB |
| GSK-3β | GSK-3b (3D10) | 9832 (CST) | Mouse | monoclonal | 1:1000 (WB) | WB |
| p-GSK-3β | Phospho-GSK-3b (Ser9) | 5558 (CST) | Rabbit | monoclonal | 1:1000 (WB) | WB |
