# Supplementary material for: miR-200c Modulates the Pathogenesis of Radiation-Induced Oral Mucositis
Source: Oxid Med Cell Longev. 2019 Jun 27;2019:2352079. doi: 10.1155/2019/2352079 (PMC6620860; doi:10.1155/2019/2352079)
Supplement: Supplementary 2 — Table S2: primer sequences for quantitative RT-PCR. [file 2352079.f2.docx]

**Table S2:** Primer sequences for quantitative RT-PCR

| Gene | **Forward primer** | **Reverse primer** |
| --- | --- | --- |
| miR-200a | GCGTAACACTGTCTGGTAACGATGT |  |
| miR-200b | GCGGTAATACTGCCTGGTAATGATGA |  |
| miR-200c | GCTAATACTGCCGGGTAATGATGGA |  |
| miR-141 | GCGTAACACTGTCTGGTAAAGATGG |  |
| has-miR-429 | GCGCGTAATACTGTCTGGTAAAACCGT |  |
| mmu-miR-429 | GCGTAATACTGTCTGGTAATGCCGT |  |
| 5S rRNA | GGGAATACCGGGTGCTGTAGGCT |  |
| mus-TNF-α | CCTGTAGCCCACGTCGTAG | GGGAGTAGACAAGGTACAACCC |
| mus-TGF-β | AGGGCTACCATGCCAACTTC | CCACGTAGTAGACGATGGGC |
| mus-IL-6 | TAGTCCTTCCTACCCCAATTTCC | TTGGTCCTTAGCCACTCCTTC |
| mus-MIP-1β | TTCCTGCTGTTTCTCTTACACCT | CTGTCTGCCTCTTTTGGTCAG |
| mus-actin | GGCTGTATTCCCCTCCATCG | CCAGTTGGTAACAATGCCATGT |
| hsa-TNF-α | TGCACTTTGGAGTGATCGGC | CTCAGCTTGAGGGTTTGCTAC |
| hsa-TGF-β | GGCCAGATCCTGTCCAAGC | GTGGGTTTCCACCATTAGCAC |
| hsa-IL-1α | AAGCAGCCATGGCAGAAGTA | GGTGGTCGGAGATTCGTAGC |
| hsa-GAPDH | GGAGTCAACGGATTTGGT | GTGATGGGATTTCCATTGAT |
