# Supplementary material for: miR-200c Modulates the Pathogenesis of Radiation-Induced Oral Mucositis
Source: Oxid Med Cell Longev. 2019 Jun 27;2019:2352079. doi: 10.1155/2019/2352079 (PMC6620860; doi:10.1155/2019/2352079)
Supplement: Supplementary 3 — Table S3: clonogenic survival parameters fitting the data to a multitarget model. [file 2352079.f3.docx]

**Table S3**. Clonogenic survival parameters fitting the data to a multitarget model.

| *D*_0_ *D*_q_ SER |  |  |  |
| --- | --- | --- | --- |
| NHK/Control 2.01 0.41 - |  |  |  |
| MHK/miR-200c 1.91 1.76 0.23 |  |  |  |

MiR-200c inhibition decreased radiorensitivity in NHK. NHK/Control and NHK/miR-200c- were cultured in 6 well plates for 24 hours, and then irradiated with the indicated doses. After 10 days, the colonies were counted. The equation of SF = 1-(1-e^-D/D0^)^N^ was applied to calculate the cellular radiosensitivity (mean lethal dose, D_0_) and the capacity for sublethal damage repair (quasithreshold dose, Dq). Those values were then used to calculate the sensitization enhancement ratio (SER). MiR-200c inhibition increased the plating efficiency of the treated cells to fold of the NHK/Control, and decreased the radiosensitivity of NHK, with a SER of 0.23.
